# Bitter Taste Receptors 38 and 46 Regulate Intestinal Peristalsis

**DOI:** 10.3390/ijms26052092

**Published:** 2025-02-27

**Authors:** Lara Camillo, Federica Pollastro, Maria Talmon, Luigia Grazia Fresu

**Affiliations:** 1 Department of Health Sciences, University of Piemonte Orientale, 28100 Novara, Italy; 2 Department of Pharmaceutical Sciences, University of Piemonte Orientale, 28100 Novara, Italy; federica.pollastro@uniupo.it (F.P.); maria.talmon@med.uniupo.it (M.T.)

**Keywords:** bitter taste receptor, gut peristalsis, inflammation, PTC, absinthin, calcium

## Abstract

Bitter taste receptors (TAS2Rs) are expressed in extraoral tissues, exerting several functions and generating a whole-body chemosensory and protective system. TAS2Rs expression has been observed in the gastrointestinal tract, although their role is poorly understood. This study aims to investigate the role of TAS2R38 and 46 in human intestinal smooth muscle cells (HISMCs) after activation with the specific bitter ligands phenylthiocarbamide and absinthin, respectively. We found that TAS2R38 and 46 activation by phenylthiocarbamide (PTC) and absinthin, respectively, induces a rapid membrane depolarization and increase of cytosolic calcium levels due to internal storage in the IP_3_ pathway, resulting in an accelerated cell contraction. Overall, this study unravels, for the first time, the contractile impact of these TAS2R subtypes on intestinal smooth muscle cells, suggesting their involvement in gut peristalsis and recommending these receptors as possible targets for new therapies.

## 1. Introduction

Bitter sensing is a key defense mechanism against potential toxic substances, orchestrated, in humans, by the large family of bitter taste receptors (TAS2Rs), composed of 25 subtypes [1,2]. Although TAS2Rs were firstly described in the oral cavity [3], evolutionary studies place them in the Cambrian period, when they exerted a crucial role in the development of herbivore–plants interactions, suggesting a primary role of TAS2Rs as nutrient sensors in the gut [4]. These observations are in line with the recent demonstration of the extraoral localization of TAS2Rs. Several studies found TAS2Rs in the respiratory airways [5,6], central nervous system [7], reproductive tract [8,9], skin [10,11], immune system [12], and gastrointestinal tract, noting that the fate of the released calcium is tissue-specific, leading to different effects on cells [13,14,15]. In several extraoral sites, TAS2Rs have been described to be involved in muscle contraction/relaxation [15,16]. The same subtype may mediate different physiopathological effects, dependently on the organ, the cell type, and the expression level [17]. For instance, in the respiratory system, TAS2R activation induces relaxation of the airway smooth muscle [18,19,20,21] but causes vasoconstriction of the pulmonary artery smooth muscle cells [22].

The variability of TAS2R effects across several tissues is strictly correlated to the signaling cascade variations, according to the anatomical localization of the receptor. Indeed, TAS2Rs are G-protein coupled receptors that, upon activation, trigger a signaling cascade partially common to all cell types and in part dependent on the cellular localization of the receptors. The common function of the downstream cascade results in an IP_3_R receptor calcium release from the endoplasmic reticulum (ER). For example, in the taste buds, the calcium rise leads to membrane depolarization, instrumental in ATP release, and the consequent activation of efferent nerve fibers for taste recognition [23], while in epithelial cells of the pulmonary system, the released calcium directly activates cilia beating [22].

In the gastrointestinal tract, TAS2Rs act as sensors of luminal stimuli, consequently activating the proper mechanical and secretory response [24]. In 2002, Wu et al. demonstrated the gene expression of several TAS2Rs in the mouse and rat gastrointestinal tract, specifically in the antral and fundic gastric mucosa, as well as in the lining of the duodenum [25]. Moreover, the chemosensory network was found in diverse cell types of the stomach epithelium and enteroendocrine cells, regulating the secretion of cholecystokinin and glucagon-like peptide-1ghrelin [26,27,28] in the intestinal tuft cells [29], whose role is the detection and elimination of intestinal parasites by inducing type II immunity [28,30]. Moreover, functional bitter taste receptors were also discovered in human and mouse gut smooth muscle cells [31], in which bitter ligands are shown to induce in vitro contraction or relaxation, depending on the gut region and the selective agonist. Interestingly Feng et al. [32] demonstrated that the loss of function of α-gustducin aggravated colitis symptoms in a mouse model, accompanied by elevated levels of TNF and IFN, confirming that bitter receptors are involved in the immune balance, inflammation, and tissue integrity of the gut. Thus, additional studies are needed to better define the role of TAS2Rs in the smooth muscle of the bowel both in both physiological and pathological conditions. For example, in case of epithelial damage, the intestinal muscle is exposed to luminal content, and the expressed TAS2Rs could sense food-derived molecules, causing overreactions such as vomiting or diarrhea. Therefore, TAS2Rs modulation could be relevant for the management of altered intestinal contraction. In this context, the study aimed to investigate whether TAS2Rs, in particular TAS2R38 and 46, are expressed in a human model of intestinal smooth muscle cells (HISMCs) and whether they can influence cell contractility.

## 2. Results

### 2.1. TAS2R38 and TAS2R46 Expression in HISMCs

**Figure 1:**
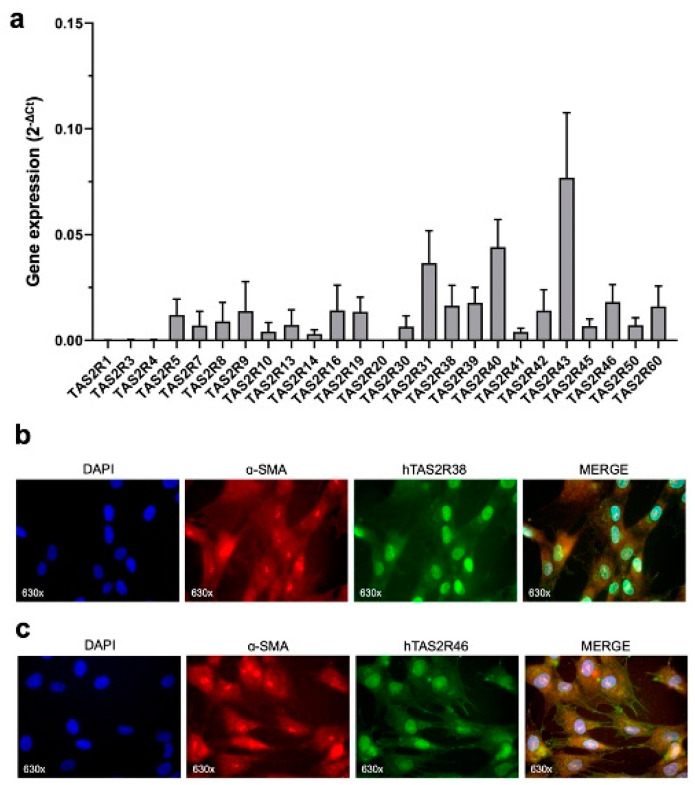
Expression of TAS2Rs in HISMC cells. (**a**) Gene expression of TAS2Rs in HISMCs represented as mean ± SEM of four independent samples. Representative images of the indirect immunofluorescence analysis of (**b**) TAS2R38 and (**c**) TAS2R46 expression in HISMCs. α-SMA was used as a marker of the cytoskeleton. Magnification: 630×.

The expression of the 25 TAS2R subtypes was evaluated by qRT-PCR to obtain a general overview of the bitter receptors in human intestinal smooth muscle cells (HISMCs). As shown in Figure 1a, most subtypes are expressed in HISMCs, including subtypes 38 and 46, the objects of this work. The expression of TAS2R38 (Figure 1b) and 46 (Figure 1c) was further confirmed through indirect immunofluorescence. We focused our analysis on these two TAS2R subtypes, despite not being more expressed, due to the increasing evidence of TAS2R38 involvement in the homeostasis and disease of several extraoral tissues, as well as due to the dilation, antioxidant, and anti-inflammatory properties of TAS2R46.

### 2.2. Activation of TAS2R38 and TAS2R46 Induces Ca^2+^ Increase in the Cytoplasm

**Figure 2:**
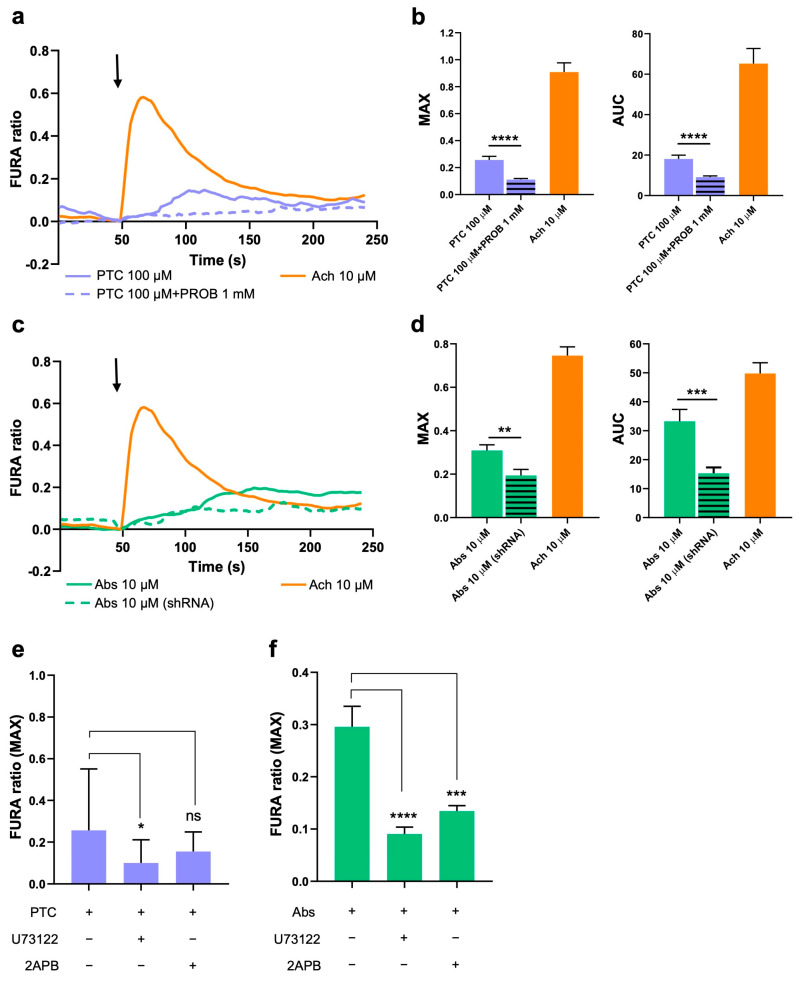
Cytosolic Ca^2+^ analysis after TAS2R38 and TAS2R46 activation: (**a**) representative FURA 2-AM traces and cytosolic Ca^2+^ after TAS2R38 activation; (**b**) maximum peak amplitude (MAX) and area under the curve (AUC) of HISMCs stimulated with 100 μM of PTC with/without TAS2R38 inhibitor (PROB 1 mM) and 10 μM of acetylcholine (Ach). Data are expressed as mean ± SEM of at least 40 cells measured in four independent experiments. **** *p* < 0.0001 vs. PTC. (**c**) Representative FURA 2-AM traces and cytosolic Ca^2+^ after TAS2R46 activation, (**d**) MAX and AUC of HISMC or TAS2R46-silenced HISMC (referred as shRNA) stimulated with 10 μM of Abs and 10 μM of Ach. Data are expressed as mean ± SEM of at least 40 cells acquired in four independent experiments. ** *p* < 0.01, *** *p* < 0.001 vs. Abs. The arrow indicates when stimuli were added. FURA 2-AM ratio of HISMCs stimulated with (**e**) 100 μM of PTC and (**f**) 10 μM of Abs in presence/absence of PLC inhibitor (U73122, 10 μM) and IP3R inhibitor (2APB, 10 μM). Data are expressed as mean ± SEM of maximum peak of at least 40 cells acquired in four independent experiments. PTC, phenylthiocarbamide; PROB, probenecid; Abs, absinthin; Ach, acetylcholine; MAX, maximum peak amplitude; AUC, area under the curve. ns *p* > 0.05; * *p* < 0.05 vs. PTC; *** *p* < 0.001 and **** *p* < 0.0001 vs. Abs.

To verify the functionality of the TAS2R38 and TAS2R46 subtypes in HISMCs, we analyzed cytosolic Ca^2+^ ([Ca^2+^]_c_) transients using a FURA 2-AM probe after the activation of both receptors using their specific agonists (Figure 2).

Acetylcholine was used as a positive control. We found that the activation of TAS2R38 with phenylthiocarbamide (PTC; 100 μM) induced a [Ca^2+^]_c_ increase, in terms of both peak amplitude (MAX) and area under the curve (AUC) (Figure 2a). To confirm that the [Ca^2+^]_c_ increase was TAS38-dependent, we co-treated the cells with probenecid, the specific antagonist for the subtype, and we observed that the effect was prevented (Figure 2a,b). Similarly, TAS2R46 activation using absinthin (Abs; 10 μM; Figure 2b,c) and Abs 100 μM (Figure S1) induced a [Ca^2+^]_c_ increase in the HISMCs. Due to the difficulty in finding a specific antagonist for TAS2R46, its expression in HISMCs was silenced by lentiviral infection (Figure S2), and the calcium rise was significantly hampered in the TAS2R46-silenced cells (referred to as shRNA).

To verify whether the [Ca^2+^]_c_ increase induced by both TAS2R38 and TAS2R46 was IP_3_-dependent, as demonstrated in other cells [33], we incubated the HISMCs with a PLC inhibitor (U73122, 10 μM) and of a IP_3_R inhibitor (2APB, 10 μM) before initiating the challenge with 100 μM of PTC (Figure 2e) or 10 and 100 μM of Abs (Figure 2f and Figure S1c). By inhibiting both PLC and IP_3_R, the [Ca^2+^]_c_ levels were significantly reduced upon activation of the receptors, confirming the direct involvement of this specific pathway, leading to ER calcium release in HISMCs.

### 2.3. TAS2R38 Activation Induces HISMCs Contraction and Membrane Depolarization

**Figure 3:**
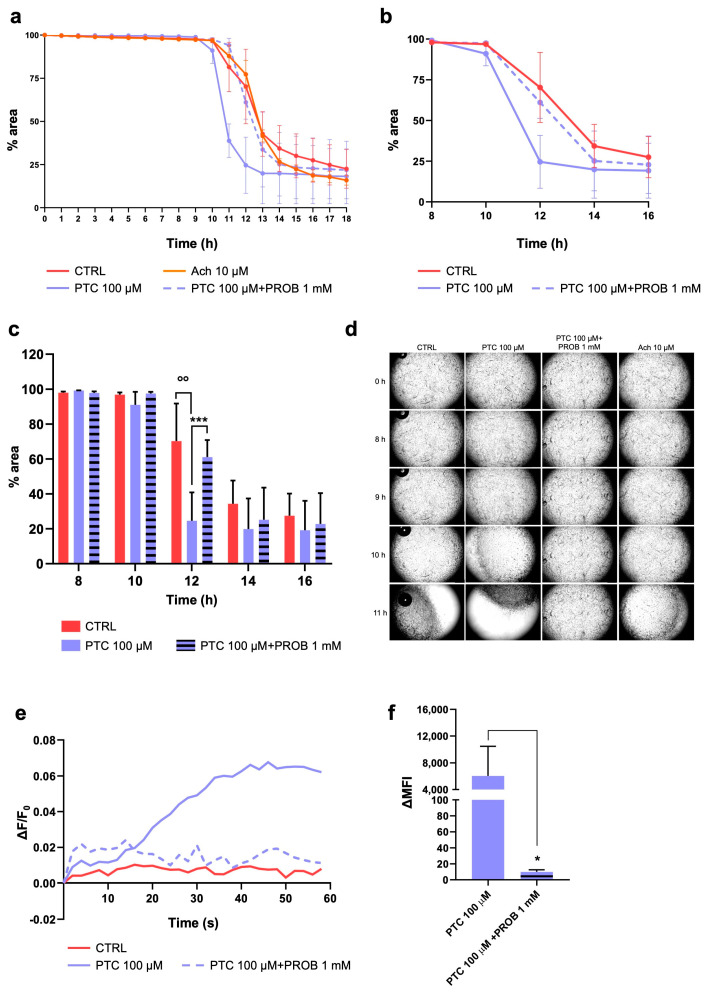
HISMC contraction and membrane depolarization after TAS2R38 activation. (**a**–**d**) HISMCs were seeded on collagen discs and treated with PTC (100 μM), with/without TAS2R38 inhibitor (PROB 1 mM). (**a**) Collagen disc areas were measured every hour, starting immediately after stimulation (0 h) until 18 h later. (**b**) Zoom view of collagen contraction 8–16 h after stimulation. (**c**) Histogram representation of zoom and (**d**) representative collagen contraction discs at 40× magnification. Data are expressed as mean ± SEM of three independent experiments. (**e**) Membrane potential analysis indicated as ΔF/F_0_ of FluoVolt fluorescence acquired with a microplate reader and (**f**) FluoVolt delta mean fluorescence intensity (ΔMFI), before and after stimulation with PTC (100 μM), with/without PROB (1 mM), measured with FACS. CTRL, no treated cells; PTC, phenylthiocarbamide; PROB, probenecid; Ach, acetylcholine; ΔMFI, delta mean fluorescence intensity. °° *p* < 0.01 vs. CTRL; * *p* < 0.05 vs. PTC; *** *p* < 0.001 vs. PTC.

We then investigated whether the increased [Ca^2+^]_c_ induced by TAS2R activation could eventually lead to HISMC contraction. We treated HISMCs with PTC (100 μM) to activate TAS2R38, with/without probenecid (PROB) (1 mM), and we evaluated the cell contraction and membrane potential changes. As shown in Figure 3, TAS2R38 activation accelerates HISMCs contraction compared to that of the control cells. Indeed, it induced cell contraction almost 9 h after treatment, reaching maximum significant contraction after 12 h (Figure 3a–c). We confirmed that the effect was TAS2R38-dependent by inhibiting the receptor with probenecid; this significantly slowed down collagen shrinkage (Figure 3c,d). To validate these results, we measured the membrane potential of HISMCs stimulated with PTC (100 μM), with/without probenecid (PROB) (1 mM), using a FluoVolt membrane potential probe (Figure 3e,f). As expected, TAS2R38 activation induced a rapid change in membrane potential, which was inhibited by probenecid. These results demonstrate that in gastrointestinal smooth muscle cells, TAS2R38 induces cell contraction by increasing [Ca^2+^]_c_ levels and membrane depolarization [34].

### 2.4. Activation of TAS2R46 Triggers Rapid Cell Contraction and Membrane Depolarization

**Figure 4:**
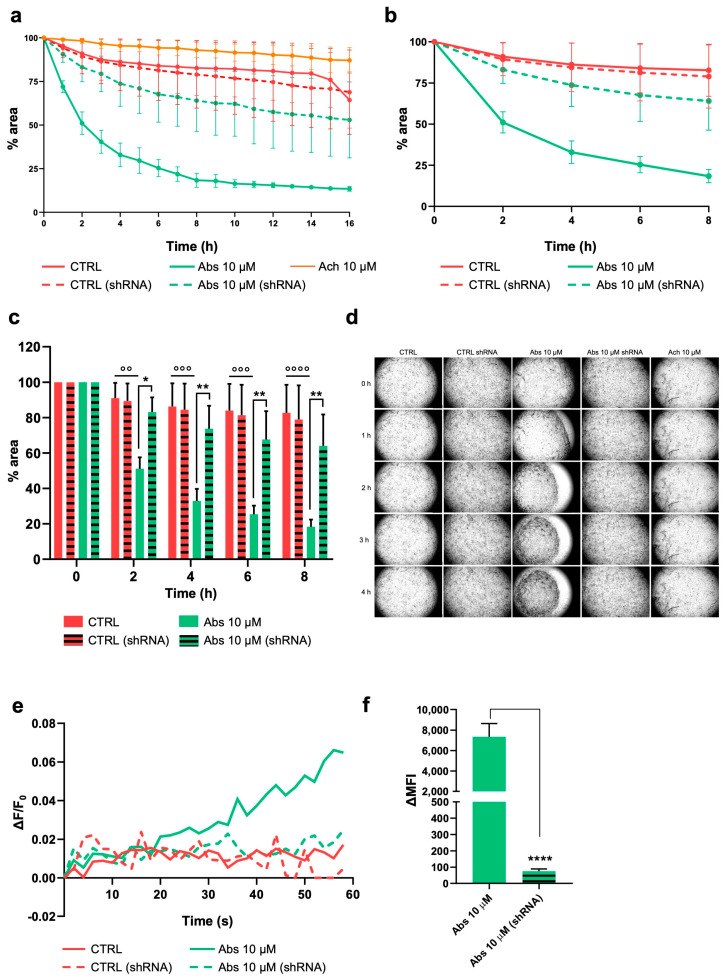
HISMC contraction and change in membrane potential after TAS2R46 activation. (**a**–**d**) HISMCs and TAS2R46-silenced HISMCs (referred to as shRNA) were seeded on a collagen disc and treated with Abs (10 μM). (**a**) Collagen disc areas were measured every hour, starting immediately after stimulation (0 h) until 16 h later. (**b**) Zoom view of collagen contraction 0–8 h after stimulation and (**c**) histogram representation of zoom. (**d**) Representative collagen contraction discs at 40× magnification. Data are expressed as mean ± SEM of three independent experiments. (**e**) Membrane potential analysis indicated as ΔF/F_0_ of FluoVolt fluorescence, acquired with a microplate reader, and (**f**) FluoVolt delta mean fluorescence intensity (ΔMFI), before and after stimulation with Abs (10 μM), measured with FACS. CTRL, untreated cells; Abs, absinthin; Ach, acetylcholine; ΔMFI, delta mean fluorescence intensity. * *p* < 0.05, ** *p* < 0.01, **** *p* < 0.0001 vs. Abs; °° *p* < 0.01, °°° *p* < 0.001, °°°° *p* < 0.0001 vs. CTRL.

Similar results were observed on HISMCs stimulated with Abs (10 μM) (Figure 4). Indeed, TAS2R46 activation induced a quick cell contraction compared with that of the control cells and those treated with acetylcholine (Ach, 10 μM) (Figure 4a). As better illustrated in Figure 4b, the collagen area started to decrease immediately after 1 h of stimulation, reaching a maximum contraction 8 h later. We can state that this effect was strictly dependent on TAS2R46 activation because in TAS2R46-silenced HISMCs (shRNA), the cell contraction was superimposable to that of the control cells (Figure 4c,d and Figure S3c,d). Again, TAS2R46 activation triggered membrane depolarization (Figure 4e,f and Figure S3e,f) that was hampered in the TAS2R46-silenced HISMC (shRNA). Thus, TAS2R46 also triggers cell contraction through an increase in [Ca^2+^]_c_ and membrane depolarization.

## 3. Discussion

Bitter perception allows for avoidance of the ingestion of potentially harmful toxic compounds through TAS2Rs present on the taste buds [4]. In recent years, their expression has been documented in several extraoral tissues, including the gastrointestinal tract, where they regulate appetite and gut hormone secretion, as well as sense harmful compounds by limiting the absorption of toxic/bitter compounds [35,36,37,38,39,40]. The majority of studies have shown that TAS2R-expressing cells use the canonical signaling pathway in response to stimulation [41]. Binding of ligands to TAS2Rs initiates a signaling cascade, leading to a dissociation of the G-protein gustducin into Gα and Gβγ subunits, followed by the activation of phospholipase C β2 (PLCβ2) [42], which promotes the production of diacylglycerol and inositol 1,4,5-trisphophate, two important mediators of calcium release. However, it is noteworthy that ectopic TAS2Rs may trigger tissue-specific signaling pathways to play different biological roles, depending on the cell types. Specifically, a few additional transduction cascades following Ca^2+^ signaling lead to muscle relaxation [19,20,43,44] and contraction [22,45]. It has been demonstrated that the loss of function of α-gustducin aggravated colitis symptoms in a mouse model, accompanied by an increase in pro-inflammatory cytokines such as TNF and IFN, confirming that bitter receptors are involved in the immune balance, inflammation, and tissue integrity of the gut [32].

Our study provides evidence that the bitter taste receptors TAS2R38 and TAS2R46 in human intestinal smooth muscle cells induce increases in cytosolic calcium and membrane depolarization, resulting in the modulation of cell contraction.

As a first observation, we demonstrated, for the first time, that HISMCs express most of the 25 TAS2R subtypes at the gene level, including TAS2R38 and 46, the objects of our study, whose expression was also confirmed at the protein level. We focused on these two subtypes for the following reasons. TAS2R38 is the most-studied subtype, and its genetic variants have been associated with several behavioral habits, e.g., smoking and diet, with an increased risk of the development of colorectal and gastric cancers [46,47,48], dysbiosis [49], and obesity [50]. Due to its interaction with potential pathogens [51], it is assumed that TAS2R38 in the intestinal tract may interact with the gut microbiota via bitter metabolites, with the bacterial quorum-sensing molecules acting as bitter ligands. Thus, TAS2R38 is indicated as relevant in the maintenance of gastrointestinal homeostasis [46,47,48]. Meanwhile, TAS2R46 is a broadly tuned receptor that has also been shown to display strong effects when stimulated by low doses of an agonist, demonstrating a high receptor sensibility [20]. Indeed, when activated by absinthin, one of its highly specific agonists, at a micromolar range of concentration, TAS2R46 mediates significant anti-inflammatory, antioxidant, and bronchial smooth muscle dilating effects [12,20]. Thus, it is reasonable to hypothesize that TAS2R46, although it exhibits a low level of expression, also represents a highly sensitive sentinel to micromolar concentrations of bitter ligands present in the gastrointestinal tract.

Furthermore, our results confirm the role of TAS2R38 and 46 in muscle contraction regulation. In particular, we demonstrated that, upon stimulation, they induce muscle contraction via PLC/IP_3_R, the common TAS2Rs signaling pathway. Indeed, we observed a cytosolic calcium increase upon treatment with specific bitter ligands, resulting in a membrane potential change, followed by muscle contraction. The results are in good agreement with those published by Avau et al. [31], who demonstrated that functional TAS2Rs were expressed on human and mouse gut smooth muscle cells, and that their activation induced cells contraction, which was efficiently counteracted by PLC/IP_3_R inhibitors.

This contractile activity induced by TAS2Rs is quite a novelty, inasmuch as several works have proved that upon stimulation, they can induce the relaxation of different types of muscle cells [52]. In fact, the activation of TAS2Rs, including TAS2R46, in airway smooth muscle (ASM) cells leads to muscle relaxation and bronchodilation [17,18,19,21,53,54]. Similarly, the bitter ligand induces relaxation in rodent and human vascular smooth muscle cells [55,56], as well as in gallbladder smooth muscle cells [57]. Recently, we demonstrated that skeletal muscle cells differentiated from urine-derived stem cells express functional TAS2R46 that, upon activation with absinthin, leads to muscle relaxation through an increase in cytosolic calcium [58]. This means that bitter receptors not only play a different role in the smooth and skeletal muscles, but also that the bitter ligands can differently affect the contractile activity of the smooth muscles, depending on the anatomical section and the function of the organ. Therefore, this work provides new insights into the effects of TAS2R activation on gut smooth muscle contractility and therefore, on intestinal homeostasis. Under physiological conditions, the intestinal peristalsis plays the specific role of digesting ingested food and eliminating waste through coordinated smooth muscle contraction [59,60,61]. The mechanism underlying intestinal peristalsis is under the control of an electrical cellular network that includes the interstitial cells of Cajal (ICC), the PDGF receptor positive cells (PDGFR)a+, the smooth muscle cells, and the enteric nervous system (ENS) [62,63]. Moreover, a relationship occurs between ENS and the central nervous system, the ENS being responsible for the local functions of the gut. This means that under physiological conditions, in the presence of an intact epithelial barrier, smooth muscle cells receive the impulse to contract from the interstitial cells, after decoding the ENS signals, and the ICCs represent a kind of pacemaker for gastrointestinal function, modulating gut motility and communicating with the enterocytes, blood vessels, and smooth muscle cells [63,64]. However, developmental failures and diseases can affect muscle contractility machinery, as occurs in IBD patients. Indeed, IBD is characterized by an altered intestinal epithelial barrier, microbiota dysbiosis, and exacerbated inflammation, resulting in abdominal pain and chronic diarrhea [65,66,67]. This abnormal intestinal motility could be reinforced by the interaction of TAS2Rs expressed on the smooth muscles, which are no longer protected by the now-damaged epithelial barrier, with quorum-sensing molecules released by the altered intestinal microbiota in response to chronic inflammation and to ingested bitter compounds [67,68,69]. Although the pathogenesis of IBD is still poorly understood, the targeting of intestinal inflammation remains the major therapeutic strategy. Unfortunately, traditional anti-inflammatory drugs, including steroids and immunosuppressants, fail in a large proportion of IBD patients, with only approximately 20% of IBD patients achieving symptom and disease control [70]. Consequently, our observation suggests that potential therapeutic modulation of TAS2Rs may induce/reduce gastrointestinal contraction and ameliorate symptoms in different disorders. This hypothesis, however, can only be confirmed through in-depth studies conducted on in vitro and in vivo models, while also taking into consideration the other TAS2R subtypes expressed in intestinal cells. In fact, as demonstrated by Avau et al. [31], in vivo oral administration of bitter ligands modulates gastric emptying time and satiation by affecting gastric smooth muscle cell movements, demonstrating a possible application in obese patients. On the other hand, bitter taste receptors expressed on tuft cells are activated by pathogen metabolites, thus triggering an orchestrated response at the gastrointestinal level by IL-25 release in order to eliminate pathogens [71,72], to repair the integrity of the gut barrier, and to reduce inflammation.

## 4. Materials and Methods

### 4.1. Cell Culture

Human intestinal smooth muscle cells (HISMCs) were purchased from ScienCell Research Laboratories (cat. #2910; Carlsbad, CA, USA) and cultured in Smooth Muscle Cell Medium (SMCM cat. #1101, ScienCell) supplemented with 2% FBS, 1% penicillin/streptomycin solution (P/S), and 1% smooth muscle cell growth supplements (SMCGS, cat. #1152; ScienCell), following manufacturer’s instructions. HISMCs from passage 3 to 10 were used for the experiments.

### 4.2. Cell Stimulation

HISMCs were stimulated alone or in combination with the following compounds, as described in the figure captions: phenylthiocarbamide (PTC 100 μM, Merck, Darmstadt, Germany), acetylcholine (Ach 10 μM, Merck), used in all the experiments as the active control, and absinthin (Abs 10 μM [20] and 100 μM). Probenecid (PROB 1 mM, Merck) was incubated for 30 min before the beginning of each experiment to allow for TAS2R38 inhibition.

### 4.3. TAS2R46 Silencing

Due to the difficulty in finding a specific antagonist for TAS2R46, its expression in the HISMCs was silenced by lentiviral infection. Two lentiviral constructs targeting human TAS2R46 (TCRN0000014110 and TCRN0000014112) were obtained from the TRCHs1.0 library (Dharmacon, Lafayette, CO, USA). Third-generation LVs were produced by co-transfecting HEK293T packaging cells with the plasmids pMDLg/pRRE, pMD2. VSVG, pRSV-Rev, and transfer construct were employed using Lipofectamine 2000 (Thermo Fisher, Waltham, MA, USA), as described previously [20]. The HISMCs were then transduced by two sequential infections, and the silencing was assessed by qRT-PCR.

### 4.4. Quantitative Real-Time PCR

The HISMCs (1 × 10^6^ cells) were resuspended in 1 mL of Trizol (Immunological Science, Rome, Italy) for total RNA extraction. The amount and purity of RNA were quantified using a spectrophotometer (Nanodrop, Thermo Fisher) by measuring the optical density at 260/280 nm. Reverse transcriptase and cDNA synthesis were performed using a SensiFAST cDNA Synthesis Kit (Bioline, London, UK), according to manufacturer’s instructions. For qRT-PCR, gene specific primers (Supplementary Table S1) and a SensiFAST SYBR No-ROX kit (Bioline) were used. Glyceraldehyde-3-phosphate dehydrogenase (GAPDH) was used as a housekeeping gene and for data normalization. Relative quantification was determined using the 2^−ΔCt^ method.

### 4.5. Indirect Immunofluorescence

The HISMCs (1.5 × 10^4^ cells) were seeded on a round sterile glass coverslip (12 mm ø), fixed in PAF 4% for 10 min at 4 °C, and incubated with blocking solution (3% BSA, 0.1% Triton in PBS) for 1 h at room temperature (RT). Then, the cells were incubated with the primary antibodies rabbit polyclonal anti-hTAS2R46 (1:500, cat# OSR00173W, RRID: AB_962255, Osenses, Australia), rabbit polyclonal anti-hTAS2R38 (1:500, cat# OST00440W, RRID: AB_3674565, Osenses), and mouse monoclonal anti-α-SMA (1:500, cat# 14976082, RRID: AB_2572996, Thermo Fisher) for 2 h at RT. The secondary antibodies goat anti-rabbit Alexa Fluor-488 (1:1000, Thermo Fisher) and goat anti-mouse Alexa Fluor-546 (1:1000, Thermo Fisher) were added along with DAPI (1:1000, Merck) for 45 min at RT in the dark. Images were captured with a fluorescent microscope, Leica DS5500B (Leica, Wetzlar, Germany), at 630× magnification.

### 4.6. Contraction Assay

The HISMCs (4 × 10^4^ cells/well) were embedded in collagen gel (collagen I, obtained from mouse tail at a final concentration of 8 mg/mL), and SMCM was added to each well, with/without stimuli (acetylcholine, 100 μM; absinthin, 10 μM; or phenylthiocarbamide, 100 μM). The cells were left to grow, and the area of collagen disk was measured by ImageJ software 1.53e (NIH, Bethesda, MD, USA) [73] every 1 h for 24 h after reading the plate using the IncuCyte S3 Live-Cell Analysis system (Sartorius, Gottinga, Germany), under 40× magnification.

### 4.7. Calcium Imaging

The HISMCs (1.5 × 10^4^ cells) were plated on a round sterile glass coverslip (24 mm ø) coated with poly-l-lysine (Merck). The day of the experiment, the cells were stimulated as previously described and loaded with 5 μm of Fura-2AM (cat# F1201, Thermo Fisher), Pluronic F-127 (0.005%; cat# P6867, Thermo Fisher) and 10 μM of sulfinpyrazone (cat# S9509, Merck) in KREB buffer (125 mM NaCl, 5 mM KCl, 1 mM Na_3_PO_4_, 1 mM MgSO_4_, 5.5 mM glucose, 20 mM HEPES, pH 7.4) for 30 min at RT in the dark. In the presence of PROB, the HISMCs were loaded with 2 μM of FURA-2AM (Thermo Fisher) and 0.04% Pluronic F-127 (Thermo Fisher), following the protocol of Wölfle et al. [74]. After washing and de-esterification (20 min), the coverslip was mounted in a chamber equipped with a thermostat and placed on the stage of a Leica epifluorescence microscope equipped with an S Fluor 40×/1.3 objective. The cells were alternatively excited at 340/380 nm by the Polichrome V (Till Photonics, Munich, Germany) monochromator, and the fluorescent signal was collected by a CCD camera (Hamamatsu, Japan) through a 510 nm band-pass filter; the images were then analyzed using MetaMorph software 7.10.4 (Molecular Devices, Sunnyvale, CA, USA) software. Basal calcium was acquired for 50 s, and then the cells were stimulated with bitter agonists, as indicated by the arrow in Figure 2 and Figure 3. To quantify the Ca^2+^ dynamics, measured as an amplitude of Ca^2+^ increase from the baseline level, the ratio values were normalized using the formula (F_i_ − F_0_)/F_0_ (referred to as normalized Fura-2 ratio).

### 4.8. Membrane Potential Analysis

Flowcytometry analysis of membrane potential was performed by loading the HISMCs (4 × 10^4^ cells) using the voltage-sensitive dye FluoVolt Membrane Potential Kit (Thermo Fisher), following manufacturer’s instructions, for 30 min at RT in the dark. The basal membrane potential was measured in 2 × 10^4^ cells using Attune NxT FACS (Thermo Fisher) with a BL1-A laser beam. The remaining 2 × 10^4^ cells were stimulated as described above, and the membrane potential was measured again using FACS. The data were expressed as delta of mean fluorescence intensity (ΔMFI), before and after stimulation.

Real-time analysis of the membrane potential was performed by loading the HISMCs (2 × 10^4^ cells/well), seeded in a 96-well microplate, with a FluoVolt probe (Thermo Fisher) for 15 min at RT in the dark. The cells were washed twice with PBS, stimulated as previously described, and obtained using a Victor X Multilabel Plate Reader (PerkinElmer, Milan, Italy), using standard FITC settings and measuring fluorescence intensity, 30 times every 2 s. The data are expressed as the ratio of the change in mean fluorescence to the initial fluorescence (ΔF/F_0_).

### 4.9. Statistical Analysis

Statistical analysis was performed using GraphPad Prism software v. 8.0.1 (GraphPad Software Inc., La Jolla, CA, USA). A two-tailed unpaired Student’s *t*-test was used to compare the two samples. The data are expressed as the mean ± SEM of “n” independent experiments, performed in triplicate, as detailed in the figure legends, and were considered significant at * *p* < 0.05.

## 5. Conclusions

This work demonstrates that bitter taste receptors TAS2R38 and 46 induce intestinal smooth muscle contraction upon their activation, suggesting their role in gut physiological and pathological conditions, thus representing new therapeutic targets for altered peristalsis.

## Data Availability

The data presented in this study are available in the manuscript.

## Supplementary Materials

The following supporting information can be downloaded at: https://www.mdpi.com/article/10.3390/ijms26052092/s1.

## Abbreviations

The following abbreviations are used in this manuscript:

Abs	absinthin
Ach	acetylcholine
HISMC	human intestinal smooth muscle cell
PROB	probenecid
PTC	phenylthiocarbamide
SMCM	smooth muscle cell medium
TAS2R	bitter taste receptor type 2
[Ca^2+^]_c_	cytosolic calcium concentration
