# Supplementary material for: Bitter Taste Receptors 38 and 46 Regulate Intestinal Peristalsis
Source: Int J Mol Sci. 2025 Feb 27;26(5):2092. doi: 10.3390/ijms26052092 (PMC11900946; doi:10.3390/ijms26052092)
Supplement: Supplementary file 1 [file ijms-26-02092-s001.zip › ijms-3477898-supplementary.pdf]

Bitter taste receptors 38 and 46 regulate intestinal peristalsis

Lara Camillo, Federica Pollastro, Maria Talmon and Luigia Grazia Fresu

Supplementary Figure S1

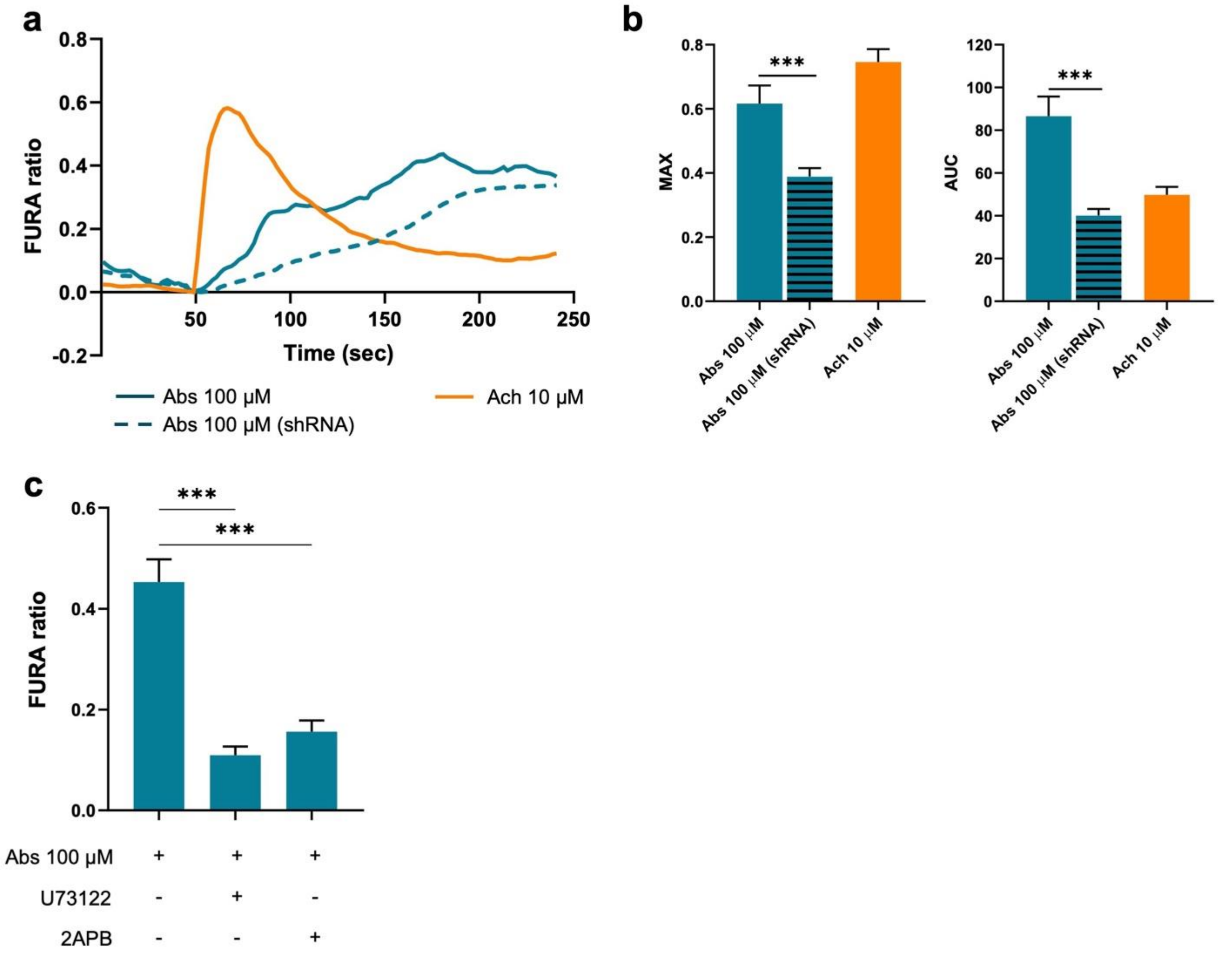

Figure S1. Cytosolic Ca^{2+} analysis after TAS2R46 activation with Abs 100 μM. **a)** representative FURA 2-AM traces and cytosolic Ca^{2+} **b)** MAX and AUC of HISMC or TAS2R46-silenced HISMC (referred as shRNA) stimulated with Abs 100 μM and Ach 10 μM. Data are expressed as mean ± SEM of at least 40 cells acquired in four independent experiments. FURA 2-AM ratio of HISMCs stimulated with **c)** Abs 100 μM in presence/absence of PLC inhibitor (U73122, 10 μM) and IP_3R inhibitor (2APB, 10 μM). Data are expressed as mean ± SEM of maximum peak of at least 40 cells acquired in four independent experiments. CTRL, no treated cells; Abs, absinthin; Ach, acetylcholine; MAX, maximum peak amplitude; AUC, area under the curve. ***p< 0.001 vs Abs 100 μM.

Supplementary Figure S2

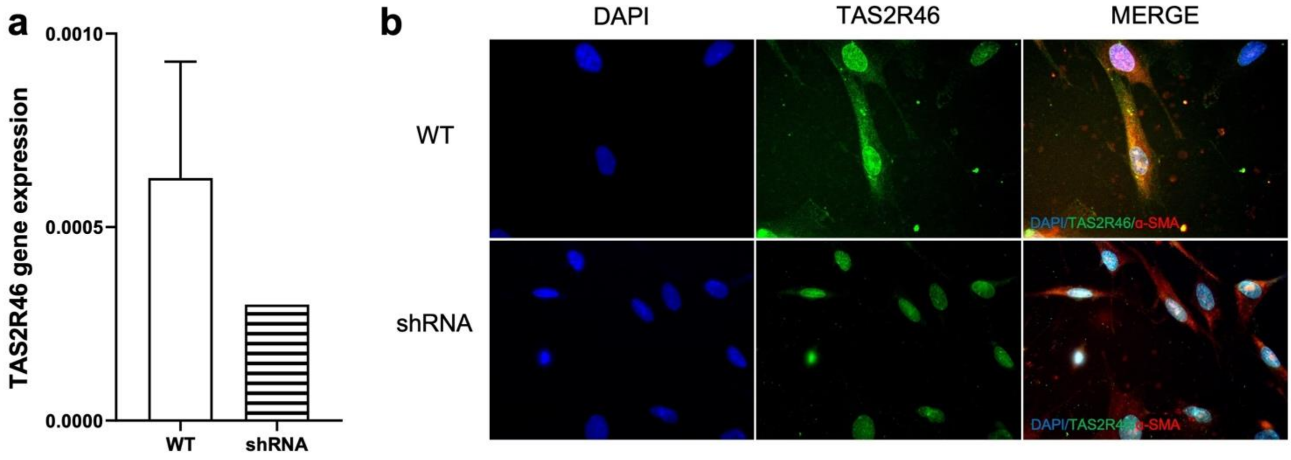

Figure S2. TAS2R46 silencing in HISMC. **a)** analysis of gene expression through qRT-PCR and **b)** indirect immunofluorescence analysis of TAS2R46 expression on HISMC (WT) and HISMC-silenced (shRNA).

Supplementary Figure S3

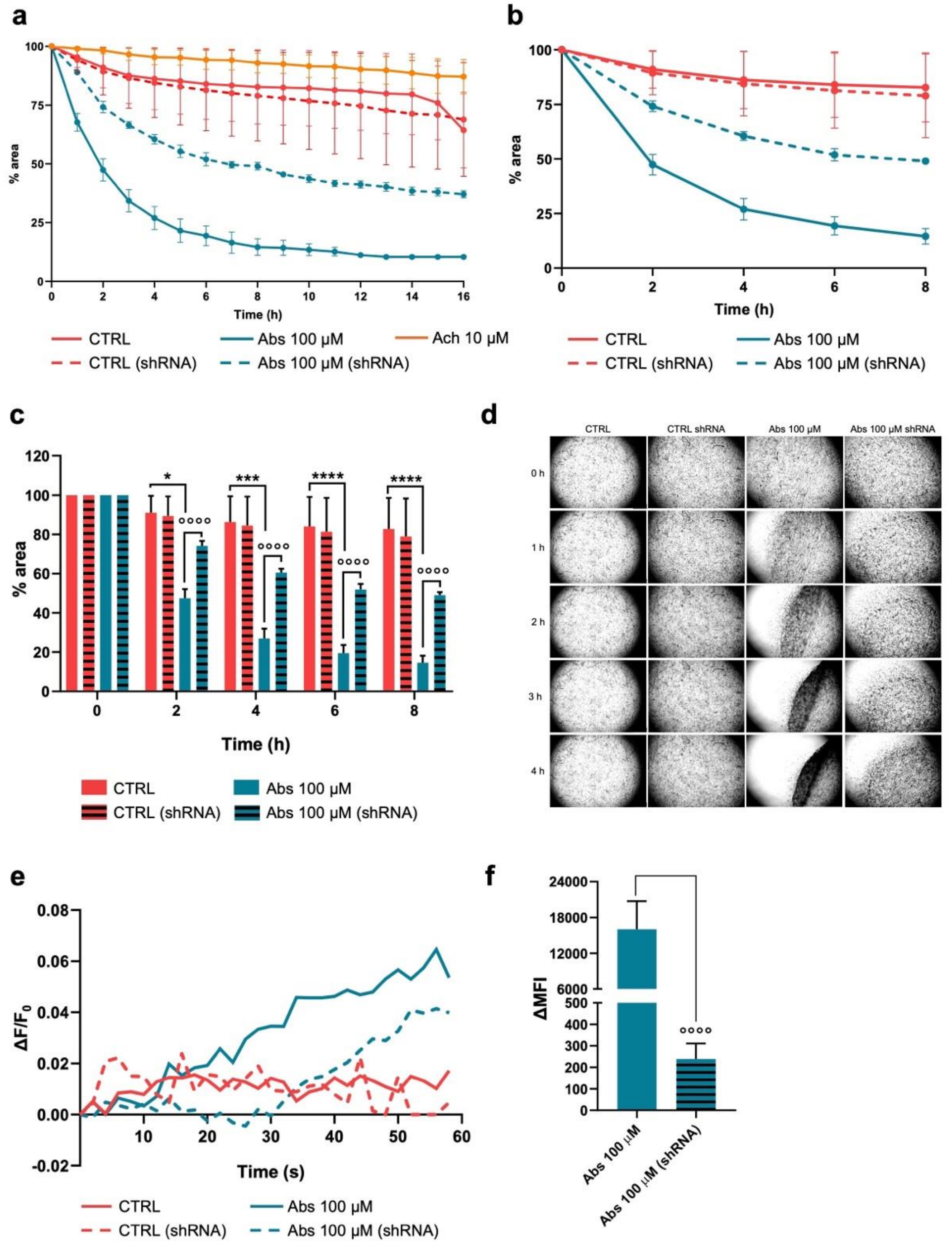

Figure S3. TAS2R46 activation induced cell contraction and change in membrane potential. **a-d)** HISMCs and TAS2R46-silenced HISMC (referred as shRNA) were seeded on collagen disc and treated with Abs 100 μM. **a)** Collagen disc areas were measured every hour starting immediately after stimulation (0h) until 16 hours later. **b)** zoom of collagen contraction between 0-8 hours after stimulation and **c)** histogram zoom representation. **d)** representative collagen contraction discs at 40x magnification. Data are expressed as mean ± SEM of three independent experiments. **e)** Membrane potential analysis indicated as ΔF/F0 of FluoVolt fluorescence acquired with micro-plate reader and **f)** Fluovolt delta mean fluorescence intensity (MFI) before and after stimulation with Abs 100 μM measured with FACS. CTRL, no treated cells; Abs, absinthin; Ach, acetylcholine; ΔMFI, delta mean fluorescence intensity. *p<0.05, ***p<0.001, ****p< 0.0001 vs CTRL; °°°°p<0.0001 vs Abs 100 μM

Supplementary Table S1

Gene name	Forward primer (5’-3’)	Reverse primer (5’-3’)
hTAS2R1	TGTGGTGGTGAATGGCATTG	TAAGAGAATTGCACAATTCGCAG
hTAS2R3	ACACATGATTCAGGGATAATAATGC	TACCATCACCCTAGAAACTCTC
hTAS2R4	TACAGTGGTCAATTGCAAAACTTGG	CTGACCTTTCCGTATTTGAAGAG
hTAS2R5	TGGTCCTCATATAACCTCATTATC	CCAGGACCCAGAAGATACTA
hTAS2R7	GTGCTATATCCAGATGTCTATGC	ATCCAGGAAATCACCCTGTC
hTAS2R8	AGTTATCGCCAGAATTTGTTTGATC	GAAGACATTAAGGCAGGTGG
hTAS2R9	TGAATTGACCATAGGGATTTGGG	AAGAGCAGCATAAAGAAGCCATC
hTAS2R10	GACTTGTAAACTGCATTGACTGTG	GCTGGTGGCAAACCACATAC
hTAS2R13	TCAGTAAAAGAGAGCTGTCCTC	CATAATTCTTAATCCTGTTCCAGAC
hTAS2R14	GCTTTGGCAATCTCTCGAATTAG	TGTCCAGATATTAGTAAGCATTCTG
hTAS2R16	CCAGGCTCATACAGTTGCAT	GAAGCGCGCTTTCATGCTT
hTAS2R19	GGTTTACTCTGGGTCATGTTATTC	GCAAACAAAATATGCTGAGGCTAG
hTAS2R20	GCACTGATAAATTTCATTGCCTGG	GAAGTTGGATTCAACACAGTTGAA
hTAS2R30	GTTATTACTACATTGGTATGCAACTC	GAGGCTAGTAGCAAGCCAGCT
hTAS2R31	GCATTGGTAAATTCCATTGAGCG	AGCTGGATTAAACACAGTTGAATAC
hTAS2R38	ACAGTGATTGTGTGCTGCTG	TGGCTTGGTAGCTGTGGTTC
hTAS2R39	TGTCGCCATTTCTCATCACCTTA	TGCCACTTGTGGAAACTGCC
hTAS2R40	GAGTGCATCACTGGCATCCTT	CAGCATCATCCAAATCTGTAGC
hTAS2R41	GGTTGCTGCCCTTGGATATGA	TACTCGACCTTCTGGGCAGA
hTAS2R42	CATTTTTTTTCCTTACAAGTGGCC	GCTTGCTGTTTCCCAGAATGAG
hTAS2R43	GCTAATGGCTTCATAGCACTGG	TGCTGAAATGGTTGATCACTGC
hTAS2R45	CTCCTTTGCTGACCAAATTGTC	AGTTGCTGAAATGGCCGGTTAC
hTAS2R46	GAGTTGAATCCAGCTTTTAACAG	GGCAATCTTGAGCAAATAAAATATGC
hTAS2R50	GGTAAATTTCATTGACTGGGTGAAGAG	GCTGGATTCAACACAGTTAAATACCAA
hTAS2R60	CAGGCAATGGCTTCATCACTG	TGGTCTTACCCATTACCACTG
GAPDH	AACGTGTCAGTGGTGGACCTG	AGTGGGTGTCGCTGTTGAAGT
